# Genetic Manipulation of Biosynthetic Pathways in Mint

**DOI:** 10.3389/fpls.2022.928178

**Published:** 2022-06-14

**Authors:** Lorenz K. Fuchs, Alistair H. Holland, Richard A. Ludlow, Ryan J. Coates, Harvey Armstrong, John A. Pickett, John L. Harwood, Simon Scofield

**Affiliations:** ^1^School of Biosciences, Cardiff University, Cardiff, United Kingdom; ^2^School of Chemistry, Cardiff University, Cardiff, United Kingdom

**Keywords:** *Mentha*, mint, terpenes, isoprenoid, metabolism, genetic manipulation, metabolic engineering

## Abstract

In recent years, the study of aromatic plants has seen an increase, with great interest from industrial, academic, and pharmaceutical industries. Among plants attracting increased attention are the *Mentha* spp. (mint), members of the Lamiaceae family. Mint essential oils comprise a diverse class of molecules known as terpenoids/isoprenoids, organic chemicals that are among the most diverse class of naturally plant derived compounds. The terpenoid profile of several *Mentha* spp. is dominated by menthol, a cyclic monoterpene with some remarkable biological properties that make it useful in the pharmaceutical, medical, cosmetic, and cleaning product industries. As the global market for *Mentha* essential oils increases, the desire to improve oil composition and yield follows. The monoterpenoid biosynthesis pathway is well characterised so metabolic engineering attempts have been made to facilitate this improvement. This review focuses on the *Mentha* spp. and attempts at altering the carbon flux through the biosynthetic pathways to increase the yield and enhance the composition of the essential oil. This includes manipulation of endogenous and heterologous biosynthetic enzymes through overexpression and RNAi suppression. Genes involved in the MEP pathway, the menthol and carvone biosynthetic pathways and transcription factors known to affect secondary metabolism will be discussed along with non-metabolic engineering approaches including environmental factors and the use of plant growth regulators.

## Introduction

In recent years, the study of aromatic plants has increased with greater interest from industrial, academic, and pharmaceutical industries, and the *Mentha* spp. (mint) are at the forefront of this interest. Plants in the genus *Mentha* are ubiquitous, found on every continent barring Antarctica (Lawrence, 2007). While peppermint was not officially described until 1696 by the English botanist John Ray and did not enter into the London Pharmacopoeia until 1721, menthol containing plants have been used since the beginning of recorded history, exemplified by the discovery of dried peppermint leaves in the pyramids of ancient Egypt (Spirling and Daniels, 2001).

**FIGURE 1:**
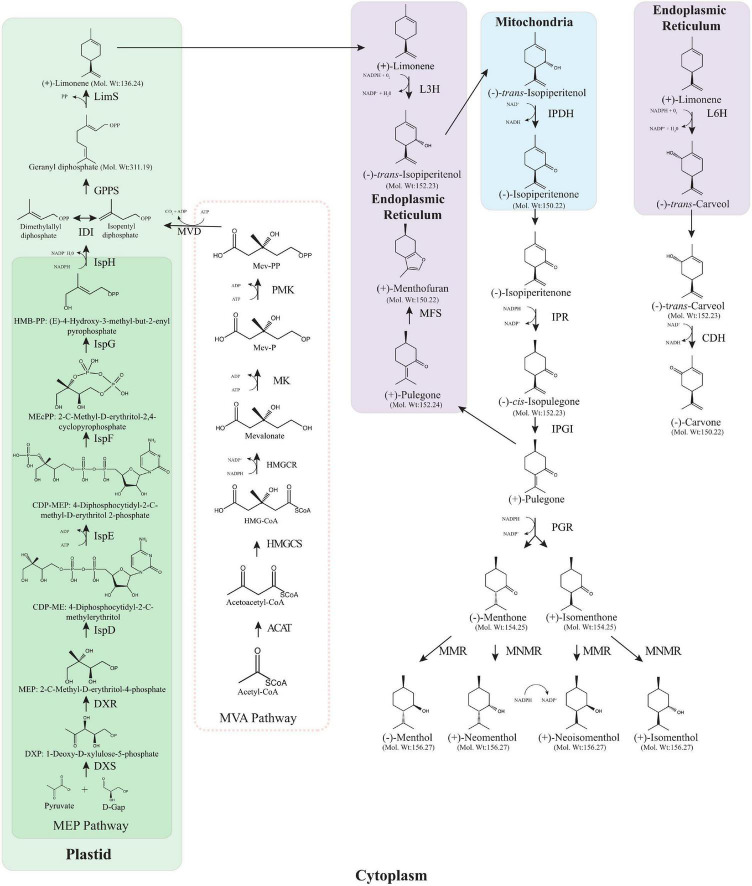
Monoterpene biosynthesis pathway in *Mentha* spp. Both the MEP and the MVA pathway are shown although the MVA pathway does not provide IPP as it is blocked in peppermint trichomes. ACAT, acetyl-coenzyme A acetyltransferases (Thiolase); HMGCS, hydroxymethylglutaryl-CoA synthase; HMGCR, 3-hydroxy-3-methylglutaryl-coenzyme A reductase; MK, mevalonate kinase; PMK, phosphomevalonate kinase; MCD, mevalonate diphosphate decarboxylase; DXS, 1-deoxy-D-xylulose 5-phosphate synthase; DXR, 1-deoxy-D-xylulose 5-phosphate reductoisomerase; IspD, 2-C-methyl-D-erythritol 4-phosphate cytidylyltransferase; IspE, 4-(cytidine 5′-diphospho)-2-C-methyl-D-erythritol kinase; IspF, 2-C-methyl-D-erythritol 2,4-cyclodiphosphate synthase; IspG, 4-hydroxy-3-methylbut-2-en-1-yl diphosphate synthase; IspH, 4-hydroxy-3-methylbut-2-enyl diphosphate reductase; IDI, isopentenyl diphosphate isomerase; GPPS, geranyl diphosphate synthase; LimS, (–)-limonene synthase; L3H, (–)-4S-limonene-3-hydroxylase; IPDH, (–)-*trans*-isopiperitenol dehydrogenase; IPR, (–)-*trans*-isopiperitenone reductase; IPGI, (+)-*cis*-isopulegone isomerase; PGR, (+)-pulegone reductase; MFS:(+)-menthofuran synthase; MMR, (–)-menthone: (–)-menthol reductase; MNMR, (–)-menthone: (–)-neomenthol reductase; L6H, (–)-4S-limonene-6-hydroxylase; CDH, (+)-*trans*-carveol dehydrogenase. The absolute stereochemistry, corresponding to the optical rotation signs, is provided structurally.

The value of *Mentha* lies primarily in the terpenoid profile of the essential oils (EO) they produce, predominantly in the form of the monoterpenoids; compounds produced in the glandular trichomes (Figure 1, showing the monoterpenes in the menthol/carvone biosynthesis pathway including stereochemistry). These profiles are what cause the widespread use as therapeutic remedies and to complement conventional therapies, as well as being the basis for indigenous and traditional healing systems that are still used worldwide. The unique monoterpenoid profile of several *Mentha* spp. is dominated by menthol, a cyclic monoterpene with some remarkable biological properties that make it useful in the pharmaceutical, medical, cosmetic and cleaning product industries (Nair, 2001; Tucker, 2006; Eftekhari et al., 2021). Essential oils from *Mentha* have proven to have interesting properties: as carminatives (May et al., 2000), antispasmodics (Heghes et al., 2019), insect repellents (Ansari et al., 2000), choleretics (Hu et al., 2015), analgesics (Yousuf et al., 2013), anti-inflammatories (Xia et al., 2021), antioxidants (Ed-Dra et al., 2020), antivirals (Minami et al., 2003), anti-tumour promoting (Ohara and Matsushia, 2002), antibacterials (Xia et al., 2021), antifungals (Piras et al., 2021), antimicrobials (Mimica-Dukic et al., 2003), anti-allergenics (Inoue et al., 2002), anti-biofilms (Fathi et al., 2021) and more recently inhibitors of SARS-CoV-2 (Jan et al., 2021). It is little wonder then that a recent market analysis showed that the global EO market was just over $10.8B USD in 2020 and this is expected to rise to over $24.7B USD in 2030 while the mint EO market was valued at $177.8M USD in 2018 and is expected to top $330 M USD by 2025 (Grand View Research, 2019; Chauhan and Deshmukh, 2022). Worldwide production of peppermint alone stood at over 48,000 tonnes in 2020 (Food and Agriculture Organization of the United Nations, 2022) and the lion’s share of this mint essential oil comes from cornmint, *Mentha arvensis*, which accounted for 60% of the revenue in the mint essential oil market in 2018 (Grand View Research, 2019).

### Taxonomy and Chemotype – Grouping of Mints

The taxonomy of the genus *Mentha* is highly complex, with more than 3,000 names having been published since 1753. Thankfully the use of phylogenetic analysis of morphology, essential oil constituents, chromosome numbers, and more recently DNA barcoding, has refined this number to include 18–24 species and 11 named hybrids (Tucker, 2012). The three most promising cultivars are *Mentha* × *piperita* (peppermint), which is primarily hexaploid but has ploidy ranging from triploid to aneuploid (Tucker and Naczi, 2007), a sterile hybrid between a tetraploid *Mentha spicata* and an octoploid *Mentha aquatica*. Mitcham peppermint arose over 250 years ago in Mitcham, England and has been clonally propagated ever since with Black Mitcham being the most commonly used cultivar for research (Tucker, 2012). The cultivated natural *M. spicata* (Spearmint) is a sterile triploid known for its characteristic scent produced by carvone (Croteau et al., 2005; Vining et al., 2020). *M. arvensis* (cornmint) also comes in a variety of ploidies but is primarily grown for its extremely high menthol concentration (Sobti, 1965). The sterility of the various mints hinders conventional breeding techniques for improving agronomic traits but other genetic mutation and recombination techniques have been undertaken to tackle this.

Mint essential oils consist predominantly of monoterpenoids, however, the compounds present vary greatly both within and between species. Indeed, many commercial cultivars are named and marketed according to their scent and not their taxon (Karousou et al., 2007). Cultivars can be clustered into specific chemotypes that are characterised by a distinct scent and subsequent uses in industry (Karousou et al., 2007). Furthermore, there are numerous instances where plants of different species share the same chemotype, by which they share the same predominant compound in their oil profile, despite being different species. One example of this phenomenon is the linalool-rich chemotype, which has been reported in *M. spicata* (Kofidis et al., 2004), *M. arvensis* (Gill et al., 1973), and *Mentha longifolia* among others (Baser et al., 1999). Studies suggest these chemotypes are closely related, despite their distinct oil profiles (Kippes et al., 2021). Subsequently, within a species of mint there are multiple chemotypes with markedly different oil profiles, which adds complexity when considering which cultivars of mint to focus efforts on to improve oil profiles through transgenic approaches.

#### Mentha × piperita

Peppermint is the primary cultivated species of mint and its oil is widely used as a flavouring or fragrance. Commonly reported chemotypes of *M.* × *piperita* consist predominantly of menthol (Schmidt et al., 2009), menthone (Ludwiczuk et al., 2016) or menthofuran (Khanuja et al., 2005). Most typically, *M.* × *piperita* essential oil consists predominantly of a mixture of menthol (20–54%), menthone (5–43%) and menthyl acetate (Kokkini, 1991; Moghaddam et al., 2013; Buleandra et al., 2016; Gupta et al., 2017). Limonene, 1,8-cineole, neomenthol and menthyl acetate are also commonly found as major oil components, but at a lower abundance.

#### Mentha spicata

*Mentha spicata* is a highly polymorphic mint and contains a wide range of chemotypes throughout the world (Shimizu and Ikeda, 1962; Lawrence, 2007). Wild-growing ecotypes of *M. spicata* in Greece were found to cluster into three distinct chemotypes. These were a group of plants which had an oil profile dominated by linalool (65.2–75.3%), another which was high in carvone (35.2–68.4%) and a third with either high piperitone oxide or piperitenone oxide (0.2–89.5% and 0.1–70.3%, respectively) (Kokkini, 1991; Kokkini et al., 2000; Kofidis et al., 2004). Similar to the second chemotype, *M. spicata* grown in the Himalayas predominantly yielded carvone (49.6–76.7%), but also contained a considerable amount of limonene (9.6–22.3%) (Chauhan et al., 2009). Again, in Turkey, two distinct chemotypes were found, both with either high carvone (68.1–80.6%) or high pulegone (44.9–49.2%) oils (Telci et al., 2004). The carvone chemotype of *M. spicata* is widely cultivated, and it appears that it has integrated with natural populations and become dominant in wild populations in many regions (Kokkini et al., 1995; Telci et al., 2004). Indeed, in an analysis of eight wild populations of *M. spicata* in China, all were of the carvone chemotype despite the wide range of area from which these plants were sampled (Zhao et al., 2013).

#### Mentha arvensis

Of the species considered here, *M. arvensis* has 3-octanol/3-octanone or high menthol chemotypes which are highly characteristic and often discriminate from other mint chemotypes in cluster analysis (Ludwiczuk et al., 2016). However, this is not reported in all regions, with North American populations of *M. arvensis*, falling into four chemotypes: Type 1 which is high in pulegone, isomenthone and menthone, Type 2 which is high in linalool and *cis*- and *trans*-ocimene, Type 3 which is high in isopulegone isomers and Type 4 which is high in *cis*- and *trans*-ocimene and in 1,8-cineol (Gill et al., 1973). Furthermore, the production of 3-octanone is not unique to *M. arvensis*, with smaller quantities produced by *Mentha japonica* (Fujita and Fujita, 1970), *M. aquatica* (Singh et al., 2020) and *Mentha pulegium* (Ouakouak et al., 2015) amongst others.

#### Mentha longifolia

The oil profile of *M. longifolia* is likewise highly variable between chemotypes. High menthol (32.5%) and menthone (20.7%) chemotypes have been reported (Hajlaoui et al., 2010). In Iran, one ecotype of *M. longifolia* was found to predominantly contain 1,8-cineole (15.6%), piperitenone oxide (15.1%) and pulegone (9.6%) and sabinene (9.5%), whilst the other contained p-mentha-3,8-diene (10.5%), 2,6-dimethyl-2,4,6-octatriene (10.1%), sabinene (7.0%), β-caryophyllene (7.0%), piperitone oxide (6.8%) and pulegone (6.6%) (Golparvar et al., 2013). A similar study on four other ecotypes in Iran later found two ecotypes rich in pulegone (33.4% and 44.8%), and two with high piperitenone oxide concentrations (26.7% and 29.1%) (Abedi et al., 2015). Indeed, there are plant populations with even higher concentrations of piperitenone oxide, such as those reported in Piedmont, Italy, which reached a concentration of 77.4% (Maffei, 1988). Carvone rich chemotypes exist too, with all isomers representing 39.3% of the total oil from plants harvested in Serbia (Džamić et al., 2010), and up to 69% of the oil in plants harvested in Hungary (Patonay et al., 2021). Patonay also reported a presumably rare chemotype containing high amounts of carvacrol, 1,8-cineole and thymol. Different again, some chemotypes contain predominantly isomenthone or menthofuran (Mimica-Dukić et al., 1991). This breadth of terpene chemistry may explain the wide range of chemotypes found in cultivars which have *M. longifolia* parentage.

#### Mentha aquatica

The oil profile of *M. aquatica* typically contains high concentrations of menthofuran, however, some atypical oil profiles have been reported (Vining et al., 2019). The oil commonly contains menthofuran, limonene, germacrene D, 1,8-cineole and β-caryophyllene as major constituents. However, the ratios of these change between chemotypes, and indeed not all plants have all these compounds in high concentrations. For instance, one chemotype contains 1,8-cineole (27.2%, menthofuran (23.2%) and β-caryophyllene (12.8%) as the three most abundant compounds (Morteza-Semnani et al., 2006). Whereas in other studies, this was instead limonene, caryophyllene and germacrene D (Malingre and Maarse, 1974), or high levels of menthofuran (up to 58.6%) (Anca-Raluca et al., 2013). A carvone rich chemotype also exists in *M. aquatica*, as well as atypical chemotypes in which over 98% of the total oil comprises compounds not mentioned above (Vining et al., 2019).

Besides the chemotype of a plant, agronomic practises, environmental conditions and extraction methods all affect the composition of the extracted oil. Clones of *M. spicata* have been shown to produce a consistent oil yield when grown in different locations (Telci et al., 2010). However, significant changes in oil composition were reported, albeit relatively minor in absolute values. The stage of crop growth also significantly alters the oil composition, with heterogeneous effects on individual components; whilst some compounds remain stable over the growing season, others rise or fall. For instance, in *M.* × *piperita* cv. ‘Kukrail,’ menthol content remains relatively stable within a range of 32.9–39.6% from 30 days after transplanting (DAT), to 180 DAT, whereas menthone increases from 8.1 to 33.8% at 150 DAT before falling to 23.6% at 180 DAT (Verma et al., 2010). Indeed, even within species, different accessions can have opposite trends in the accumulation/loss of specific chemicals throughout the growing season (Llorens-Molina et al., 2020). Water deficit significantly reduces oil yield and alters the oil profile in *M. spicata*, highlighting the need for controlled growth conditions when comparing the oil of mint cultivars (Okwany et al., 2012). Seasonal variation is reported in overall oil yields (Neugebauerová and Kaffková, 2013; Salim et al., 2014), as well as significant affects from fertiliser application and time of planting (Piccaglia et al., 1993; Soltanbeigi et al., 2021). Evidence also suggests that rhizobacteria such as *Pseudomonas fluorescens*, *Bacillus subtilis*, and *Azospirillum brasilense* upregulate pulegone and menthone biosynthesis, and so the microbial communities of the fields in which the mint is grown are also relevant considerations. Oil profiles also vary significantly under different extraction methods (Dai et al., 2010; Rodríguez-Solana et al., 2015).

### Monoterpenoid Biosynthesis

The menthol biosynthesis pathway (Figure 1) has been well studied over the years with only *cis*-isopulegone isomerase (IPGI) yet to be characterised in *Mentha*. The storage and biosynthesis of essential oils in *Mentha* is constrained to the peltate glandular trichomes, these oil glands are located on the aerial surfaces of the plant. From primary metabolism to menthol requires eight enzymatic steps, beginning with the parent olefin limonene formed by limonene synthase (LimS) through the cyclisation of the universal monoterpene precursor geranyl diphosphate. Geranyl diphosphate is formed by geranyl diphosphate synthase (GPPS) using the universal C_5_ isoprenoid precursors dimethylallyl diphosphate (DMAPP) and isopentyl diphosphate (IPP) derived from the methylerythritol phosphate (MEP) pathway (Croteau et al., 2005). The next step is the formation of *trans*-isopiperitenol where limonene is transferred to the endoplasmic reticulum (ER) and undergoes an O_2_ and NADPH dependent hydroxylation by limonene-3-hydroxylase (L3H). *trans*-isopiperitenol is transferred to the mitochondria where it undergoes allylic oxidation to form isopiperitenone catalysed by the NAD-dependent *trans*-isopiperitenol dehydrogenase (IPDH). Following this, the remaining steps take place in the cytoplasm, starting with the reduction of isopiperitenone to *cis*-isopulegone by isopiperitenone reductase (IPR). IPGI conducts the fifth step in the pathway by moving the double bond of *cis*-isopulegone into conjunction with the C3 carbonyl to produce pulegone. From here there are two branch points on the menthol biosynthesis pathway, one leading eventually to menthol and its seven stereoisomers, and the other leading to menthofuran. Menthofuran synthase (MFS) transforms pulegone to menthofuran in the ER (Battaile and Loomis, 1961). Pulegone reductase catalyses the conjugation of the double bond of pulegone to produce both menthone and isomenthone (Ringer et al., 2003). The final step involves the reduction of menthone to menthol which is done by one of two distinct reductases that act on menthone and isomenthone (Croteau and Gershenzon, 1994). Menthone:menthol reductase (MMR) operates on menthone to form menthol and on isomenthone to form neoisomenthol while menthone:neomenthol reductase (MNMR) operates on menthone to produce neomenthol and on isomenthone to produce isomenthol (Croteau et al., 2005).

The carvone biosynthesis pathway (Figure 1) has fewer steps than the menthol pathway but follows the same initial trajectory up until limonene. Rather than limonene undergoing C3 hydroxylation, limonene-6-hydroxylase (L6H) mediates the C6 hydroxylation in the ER to *trans*-carveol in a step toward carvone production (Karp et al., 1990). *trans*-carveol is oxidised to carvone, the main constituent of spearmint essential oil, by *trans*-carveol dehydrogenase (Turner and Croteau, 2004). A recent draft sequence of the *M. longifolia*, a diploid ancestor of *M. spicata*, has proved a valuable resource for molecular breeding and metabolic engineering (Vining et al., 2019). This was illustrated through the use of the draft genome to characterise several promoters from peppermint, known to drive expression of genes in glandular trichomes; *LimS*, *L3H*, *ISPR*, and *MMR*. Generating β-glucuronidase (GUS) constructs driven by these promoters revealed the promoter specificity to the glandular trichomes in peppermint and to non-glandular trichomes in *Arabidopsis thaliana* (Vining et al., 2017). As some enzymes such as limonene synthase, pulegone reductase and menthone-menthol reductase show temporal or developmental stage specific expression (McConkey et al., 2000), the use of gene specific promoters could be used to tailor the expression of enzymes to the areas where they can be utilised the most. The level of characterisation of both biosynthetic pathways coupled with the draft genome sequence of *M. longifolia* has opened the door for metabolic engineering to create more desirable essential oil compositions within the *Mentha* spp.

The high commercial value of *Mentha* essential oils make metabolic engineering of its biosynthetic pathways extremely lucrative for commercial growers and smallholders alike. The biosynthesis of monoterpenes in *Mentha* spp. has been well characterised by the work of Lange, Croteau and many others. Numerous studies have provided promising results demonstrating the utility of using metabolic engineering approaches to target the enzymes involved in monoterpene biosynthesis in ways that can increase the yield and improve the composition of the oil. Whilst reviews exist that range from the metabolic engineering of terpenoids in plants to the pharmacological, toxicological and insecticidal effects of *Mentha* spp., this review focuses on metabolic, environmental and plant growth regulator (PGR) modification attempts to manipulate the terpenoid content/yield done solely in *Mentha* spp. (McKay and Blumberg, 2006; Kumar et al., 2011; Malekmohammad et al., 2019).

## Engineering Monoterpenoid Biosynthesis

Despite their differences in EO, plants belonging to the *Mentha* genus employ the same enzymatic pathways and anatomical structures for the production and storage of EO. The site of this synthesis and storage is localised to the peltate (shield shaped) glandular trichomes that develop on the surface of mint leaves, mainly on the abaxial side (Turner et al., 2000b). Glandular trichomes, while functionally and morphologically dissimilar to capitate and non-glandular trichomes, are not unique to the *Mentha* genus, but are widespread across isoprenoid producing higher plant species (Turner et al., 2000b; Booth et al., 2017; Huchelmann et al., 2017). The development and distribution of peltate glandular trichomes on peppermint has been extensively characterised and for detailed descriptions see Maffei et al. (1989) and Turner et al. (2000a,b). Glandular trichomes in *Mentha* spp. are thought to be putatively involved in herbivory plant defence (Lange and Ahkami, 2013), but this is yet to be confirmed and it is entirely possible they perform a different function(s). The peltate glandular trichomes in *Mentha* consist of nine cells attached to the leaf epidermis *via* a basal cell, eight secretory cells are arranged in a disc shape and on top of these there is a large subcuticular structure that stores the isoprenoid compounds secreted from the secretory cells. Observations have shown that from initial development to the point at which trichomes are filled with EO takes over 60 h (Turner et al., 2000b; Croteau et al., 2005), and that younger leaves possess a higher density of glandular trichomes. This is unsurprising considering that the young mint plant’s metabolism is directed toward growth as it competes for canopy and root space while the older plant can focus its metabolism toward secondary metabolite production. These studies indicate that harvesting mint plants should be done when the plant is fully mature to maximise EO yield and that leaf age is an important factor when measuring trichome density or EO yield.

Scientific consensus was that the IPP and DMAPP for menthol biosynthesis was supplied by the mevalonic acid pathway (MVA), but the use of radiolabelling has shown that the IPP and DMAPP precursors are exclusively supplied by the methylerythritol phosphate (MEP) pathway (Eisenreich et al., 1997; Lange and Croteau, 1999). The MEP pathway is found in plants, bacteria and algae (Lohr et al., 2012) and operates solely in the chloroplast whereas the MVA pathway functions only in the cytosol (Lange and Croteau, 1999). The first enzyme toward menthol biosynthesis is 1-deoxy-D-xylulose 5-phosphate synthase (DXS) and this appears to be a sensible target for upregulation and while studies done in *Lycopersicon esculentum*, Ginkgo biloba and *Arabidopsis* resulted in increases in terpenoid biosynthesis (Vaccaro et al., 2014; Ikram et al., 2015); this was not the case when upregulated in peppermint (Lange et al., 2011). However, targetting the second MEP pathway enzyme has produced significant increases in EO and menthol yields. Mahmoud and Croteau (2001) transformed *M.* × *piperita* with the native *1-deoxy-D-xylulose 5-phosphate reductoisomerase* (*DXR*) gene, driven by the CaMV 35S promoter and found a significant increase in EO yield by almost 1.5 fold compared to the WT control. This was subsequently reiterated in a separate study that showed a 44% EO yield increase in *M.* × *piperita* when overexpressing *DXR* with the CaMV 35S promoter (Lange et al., 2011). Lange et al. (2011) also combined this *DXR* overexpressing line with an *MFS* RNAi line, discussed below.

The next stage of the menthol biosynthesis pathway in *Mentha*, shown in Figure 1, has been well characterised and begins with geranyl diphosphate. This pathway has been extensively reviewed in Croteau et al. (2005). Prenyltransferases are responsible for the formation of geranyl diphosphate, farnesyl diphosphate and geranylgeranyl diphosphate from the C_5_ subunits to form the C_10_, C_15_, and C_20_ precursors of monoterpenes, sesquiterpenes and diterpenes, respectively. With geranyl diphosphate being the universal precursor for monoterpenes it is a logical target for gene expression manipulation. GPPS was first characterised in 1999 by Burke, Wildung and Croteau where they discovered that MpGPPS is heteromeric, consisting of a small subunit, SSU and a large subunit, LSU, both containing an N-terminal plastid localisation signal peptide, in fact the majority of monoterpene synthases thus far cloned have plastid targetting sequences and some have been shown to localise to the plastids (Haudenschild and Croteau, 1998; Burke et al., 1999; Turner et al., 1999). They observed no prenyltransferase activity when each cDNA was expressed individually, only co-expression produced a functional GPPS (Burke et al., 1999). Heteromeric GPPSs have only been described in angiosperms known for their production of large quantities of monoterpenes in specific organs such as flower petals and trichomes (Tholl et al., 2004; Wang and Dixon, 2009). Unsurprisingly, it was found that the protein sequences of both the LSU and the SSU resembled existing prenyltransferases with the LSU sharing 62–75% identity with homodimeric geranylgeranyl diphosphate synthase (GGPPS) and 25% identity with farnesyl diphosphate synthase (FPPS), whereas the SSU had only 25 and 17% identity to GGPPS and FPPS, respectively. As both GGPPS and GPPS are plastidal this led the authors to suggest that the evolutionary origin of the LSU and SSU is from the GGPPS and not the cytosolic FPPS (Burke et al., 1999).

Further studies identified the ability of MpGPPS.SSU to modify the chain length specificity of GGPPS to favour the production of the C_10_ monoterpene carbon chains (Burke and Croteau, 2002). Co-expression of the phylogenetically distant *GPPS* from *Abies grandis* and *Taxus canadensis*, both of which produce homodimers, with the *MpGPPS.SSU* in *E. coli* resulted in the formation of functional hybrid heterodimers that were capable of producing geranyl diphosphate (Burke and Croteau, 2002). However, their results showed that the MpGPPS.SSU was unable to modify the chain length specificity of FPPS. This could be due to the plastid localisation sequences found in the GGPPS and GPPS, whereas FPPS is cytosolic.

The MpGPPS.LSU is inactive alone, whereas GPPS.LSU from *Antirrhinum majus* is capable of converting the C_5_ DMAPP and IPP into the C_20_ geranylgeranyl diphosphate *in vitro* (Burke and Croteau, 2002; Tholl et al., 2004). The ability to modify the chain length specificity of GGPPS is not limited to MpGPPS.SSU. The *A. majus* GPPS.SSU is also capable of modifying the chain length specificity of GGPPS, and when transformed into *Nicotiana tabacum* it resulted in an increase in monoterpene emission due to the augmented GPPS activity, showing that the catalytically inactive AmGPPS.SSU was able to form an active GPPS with the endogenous LSU partner *in planta* (Orlova et al., 2009). Efforts have been made to create a heteromeric GPPS in mint; Burke et al. (2004) fused the MpGPPS.SSU and the MpGPPS.LSU with a 10 amino acid linker and the resultant fusion protein resembled the kinetics, architecture and product chain-length specificity of native heteromeric enzymes, proving a suitable replacement for biological purposes when a single gene transcript is required (Burke et al., 2004). Other attempts to increase *GPPS* expression have been done using the homodimeric *GPPS* from *A. grandis*. Lange et al. (2011) found that some of their transgenic peppermint lines had increases in oil yield of up to 18% over WT controls. One of their transgenic lines, with high expression levels of *AgGPPS*, also had desirable effects on the oil composition with <2% pulegone and <5% menthofuran (Lange et al., 2011). The latest study on the efficacy of the MpGPPS.SSU on increasing monoterpene production was done by Yin et al. (2017) in *N. tabacum* and *Nicotiana benthamiana*. They co-expressed *PaLimS* with several monoterpene synthases, *MpGPPS.SSU*, *AtGPPS*, and *SlDXS*, in a transient assay using *N. benthamiana* to determine their effects on limonene. Neither the combination of *AtGPPS* or *SlDXS* with *PaLimS* showed any significant changes in limonene concentration but a 22–35 fold increase was observed with co-expression of *MpGPPS.SSU*, compared to the control plants expressing *PaLimS* alone. This suggests that neither overexpression of *SlDXS* or *AtGPPS* is capable of redirecting the metabolic flux from geranylgeranyl diphosphate to geranyl diphosphate in tobacco plants. Interestingly, these results are inconsistent with earlier studies done by Wu et al. (2006) and Hsieh et al. (2011) who found that co-expression of *LimS* and *AtGPPS* resulted in a 10 fold increase in limonene production and showed that AtGPPS seems to be multi-functional and catalyses the production of long chain prenyl diphosphate over geranyl diphosphate, respectively.

Following this the ability of MpGPPS.SSU to increase the production of other monoterpenes, *Picea sitchensis* (–)-pinene synthase (PsPinS), *Picea abies* myrcene synthase (PaMyrS) and *P. abies* linalool synthase (PaLinS), was tested. Again, the results showed an increase in each monoterpene studied and while the fold changes were not as large as those seen with limonene production, they show that MpGPPS.SSU can direct the metabolic flux toward geranyl diphosphate in *N. benthamiana* leaves. Using these successful preliminary results several transgenic *N. tabacum* plants were generated that contained *PaLinS*, *PaLimS*, *PsPinS*, and *PaMyrS* with or without co-expression of *MpGPPS.SSU*. The co-expression of *MpGPPS* and *PaLimS* resulted in a 4–8 fold increase in limonene production. Some of the other enzymes co-expressed with MpGPPS.SSU were equally as impressive with a 6–19 fold increase in linalool production, a 2–14 fold increase in myrcene production and a 2–4 fold increase in a-pinene/b-pinene production. Yin et al.’s (2017) results revealed the capability of MpGPPS.SSU to enhance various monoterpene production pathways, both transiently and stably, in *N. benthamiana* and *N. tabacum*, respectively.

Geranyl diphosphate is not just a monoterpenoid precursor, it is essential in geranylgeranyl diphosphate production by GGPPS as it is an allylic co-substrate with IPP for geranylgeranyl diphosphate. When Orlova et al. (2009) transformed *N. tabacum* with *AmGPPS.SSU* they observed an increase in endogenous monoterpene production by modification of the GGPPS product specificity, resulting in a reduction of GGPP-derived metabolites including carotenoids, GA_3_ and chlorophyll and these transgenics showed signs of adverse effects such as leaf chlorosis and dwarfism. Yin et al. (2017) observed different phenotypes in transgenic tobacco expressing *MpGPPS.SSU* including an increase in axillary buds, cytokinins, *trans*-zeatin and N6-(Δ2-isopentenyl)adenine were increased around threefold, faster growth rates and early flowering were observed, but there was no reduction in Ch1 levels between transgenic and WT plants. Upon further investigation it was found that two of the four *GGPPS* candidates in tobacco were upregulated, suggesting that MpGPPS.SSU affected the expression of *GGPPS3* and *GGPPS4* in some way, meaning it could work to increase both geranyl diphosphate and geranylgeranyl diphosphate for GA_3_ and monoterpene biosynthesis. In contrast the snapdragon GPPS small subunit was able to form a functional GPS through interaction with GGPPS1 and GGPPS2, resulting in the reduction of geranylgeranyl diphosphate and its derivatives (Orlova et al., 2009; Yin et al., 2017).

The majority of geranyl diphosphate in *Mentha* is cyclised to form limonene (Figure 1) which represents the first committed step in the biosynthetic pathway of menthol and carvone. LimS facilitates the formation of limonene, a monocyclic monoterpene, by catalysing the stereo specific cyclisation of geranyl diphosphate (Colby et al., 1993). First characterised by Rajaonarivony et al. (1992) and found to contain an N-terminal transit peptide that targets the plastids causing localisation to the leucoplasts of oil gland secretory cells in peppermint (Turner et al., 1999), LimS has been suggested as a rate limiting step for monoterpenoid production (Gershenzon and Croteau, 1990). The first attempt to alter the expression of *LimS* was done by transforming the *M. spicata LimS* gene into *M.* × *piperita* (Krasnyanski et al., 1999). When transforming protoplast-derived calli and internode sections using PEG mediated direct gene transfer and *Agrobacterium* transformation of internodal sections, respectively, their results showed that the transgenic plants had a similar profile to the non-transformed control. However, when compared to a typical mid-west piperita the transgenics had reduced menthol and increased menthone, menthofuran and pulegone contents. As single-plant data may not provide an accurate picture of the plant’s chemotype, the authors suggest the different oil profiles could be down to the use of hydrodistillation in their study compared to the use of steam distillation for the extraction of the mid-west piperita. The use of direct gene transfer resulted in multiple insertions and rearrangements as plants were found that were positive for the kanamycin resistance gene but were lacking the *MsLimS* gene. Interestingly this was not the case for the *Agrobacterium* transformed plants which exhibited single insertions. This was the first identification of this phenomenon in peppermint but tallies with previous work done in rice (Dong et al., 1996).

Following this work, Diemer et al. (2001) transformed *MsLimS* into *M.* × *piperita* and *M. arvensis*. The four transgenic *M.* × *piperita* exhibited increased total monoterpene contents compared to the wild type controls and the two *M. arvensis* plants showed decreased total monoterpene contents. Secondly, they tested quantities of the products of other enzymes that also use geranyl diphosphate as a substrate to see if there was any competition from increased LimS levels. Some of the *M.* × *piperita* plants exhibited increased (74% increase on WT) and others exhibited decreased (22% of WT) cineole levels. One *M. arvensis* plant had very high levels of ocimenes but neither *M.* × *piperita* nor *M. arvensis* plants showed changes in sabinene levels. Three *M.* × *piperita* and one *M. arvensis* plants exhibited increased pulegone levels, from 18 to 40 times the control plant, and the *M. arvensis* plant also had unusually high levels of piperitone and isopiperitenone, usually minor components of *M. arvensis* oil, and a complete absence of menthol and menthone. It was speculated, and quite likely, that the *M. arvensis* transgenic plants with decreased total monoterpenoid content and lack of menthol and menthone was due to the presence of at least five truncated copies of the *MsLimS* gene found in the plant. These opposite effects on terpene levels seen in *M.* × *piperita* and *M. arvensis* are not unheard of and have even been seen when introducing a gene into the same species (Bavage et al., 1997).

To modify the metabolic engineering of monoterpene biosynthesis through *LimS* expression it seems that the subcellular localisation is an important factor to consider. Ohara et al. (2003) found that targetting LimS to the plastids in tobacco plants gave a threefold increase in limonene production compared to the plants with cytosolic localised LimS, while targetting LimS to the ER resulted in no detectable enzyme activity (Ohara et al., 2003). Further studies showed that the low limonene production of the cytosol targetted LimS can be counteracted by co-expressing a cytosolically targetted form of GPPS, resulting in a sixfold increase in limonene production compared to the cytosolically targetted LimS alone (Wu et al., 2006). This is however, dwarfed by the 10 fold increase in limonene production resulting from targetting both GPPS and LimS to the plastids of the tobacco cells (Wu et al., 2006). Another study using the CaMV 35S viral promoter found that the constitutive overexpression, while only moderate, was insufficient to increase the production of LimS in the glandular trichomes and thus had no influence on the oil yield or composition in the transformed plants (Mahmoud et al., 2004). The authors suggested that there may be a selection pressure against the plants that show high ectopic expression of LimS as production of monoterpenes in non-specialised cells could be toxic due to the high levels of limonene that have been demonstrated to be lethal to plant tissues (Brown et al., 1987).

As plants of the *Mentha* spp. are known to be producers and stores of large amounts of monoterpenes, Li et al. (2020) attempted to use these natural facilities to produce other terpenes. Using *Mentha spicata* (known for its production of limonene and carvone) they reduced the expression of the endogenous *MsLimS* through RNAi. Their resulting transgenics showed significant reductions in limonene production (65–98%) and carvone production (67–91%) but increases in sesquiterpenes (38–96%), fatty acids (40–44%) and in flavonoids and phenolic metabolites (65–85%) when compared to the WT (Li et al., 2020). Taking this further they introduced three heterologous terpene synthases into their *MsLimS* RNAi lines, myrcene synthase and linalool synthase from *P. abies* and geraniol synthase from *Cananga odorata*. Neither the *LimS* RNAi lines nor any subsequent line harbouring the exogenous terpene synthase genes showed any phenotypic changes.

Limonene can be hydroxylated by different cytochrome P450 hydroxylases to produce characteristic regiospecific oxygenation patterns (Lupien et al., 1999). In peppermint, limonene-3-hydroxylase (L3H) specifically hydroxylates the C3 position of LimS to form *trans*-isopiperitenol (Figure 1) while in spearmint L6H specifically hydroxylates the C6 position to produce *trans*-carveol, which is further oxidised by carveol dehydrogenase to carvone (Turner and Croteau, 2004).

Mahmoud et al. (2004) used the CaMV 35S promoter to overexpress the *L3H* gene in Black Mitcham and found that the strong constitutive expression led to a high rate of co-suppression (>70%) and an accumulation of limonene, up to 80% compared with 2% in the WT, without an influence on the yield. These were the first experiments to demonstrate that the altered expression of *L3H* leads to the replacement of the normal C3 oxygenated monoterpenes menthol, pulegone, menthone and menthofuran with limonene, an olefin with vastly different volatility and polarity. Remarkably this is managed by the existing secretion and glandular trafficking machinery with no effect on the overall yield, this is promising for the potential to exploit Mentha oil glands to produce a range of hydrophobic compounds not normally accumulated in non-secretory plants due to their toxicity. Two allelic variants have been identified in *M.* × *piperita* (PM2 and PM17) which are 93% identical at the nucleotide and amino acid levels (Lupien et al., 1999). L3H isolated from *M. spicata* L. ‘Crispa’ showed more homology to the L3H’s from *M.* × *piperita* (88 and 95%) than to the L6H from *M. spicata* (71% identity) (Lupien et al., 1999; Lücker et al., 2004). Lücker et al. (2004) transformed this *L3H* into transgenic tobacco plants, engineered to produce and emit γ-terpinene, β-pinene and limonene, and found that some of the transgenic tobacco exhibited lower levels of these products. As these transgenes were driven by the CaMV 35S promoter they suggest that this could be due to gene silencing caused by multiple copies. As LimS and L3H are localised to different cell compartments their results are a promising indicator that the hypothesised transport mechanism, active or passive, for monoterpenes to the ER likely functions in tobacco and could be common in plants (Turner and Croteau, 2004). This, coupled with the use of a range of promoters may prove useful in the future of metabolic engineering of monoterpene biosynthesis.

Remarkably little has been investigated in the biosynthetic steps between limonene and pulegone. IPDH, IPR, and PR have been identified and characterised in peppermint but IPGI remains elusive (Ringer et al., 2003, 2005; Currin et al., 2018). One group was able to identify a Δ5-3-ketosteroid isomerase from *Pseudomonas putida* capable of acting as an IPGI and producing pulegone from *cis*-isopulegone. By engineering this bacterial Δ5-3-ketosteroid isomerase they improved its activity resulting in a 4.3 fold increase in IPGI activity in their *in vitro* trials in *E. coli* (Currin et al., 2018), highlighting the potential of microbial hosts for monoterpene production from inexpensive precursors.

When traversing the menthol biosynthesis pathway and arriving at pulegone, a central intermediate, there are two options for this branch point metabolite. It may be oxidised to menthofuran or reduced to menthone (Figure 1). The quantity of menthofuran in the essential oil of *Mentha* spp. is often considered undesirable as it can give off an unpalatable scent (Benn, 1998) so there have been several attempts to reduce its prevalence in the extracted essential oil. First characterised in 2001, menthofuran is a diversion from the menthol pathway through a transformation of pulegone to menthofuran by MFS (Bertea et al., 2001). MFS and L3H bear some resemblance to cytochrome P450 oxygenases that are involved in phenylpropanoid biosynthesis and both are localised to the endoplasmic reticulum (Bertea et al., 2001). Menthofuran is often considered a stress metabolite due to its increased production during water and nutrient deficiency, short day lengths and poor light intensities (Burbott and Loomis, 1967; Clark and Menary, 1980; Spencer et al., 1993). The first instance of metabolic engineering to modify MFS levels was a combination effort previously mentioned (Mahmoud and Croteau, 2001), where they upregulated the expression of *DXR* and downregulated the expression of *MFS* thorough antisense expression. Four of the transgenic plants accumulated 35–55% less menthofuran and 40–60% less pulegone with substantially more menthol than the WT controls (Mahmoud and Croteau, 2001). One transgenic line (designated MFS7A) consistently produced an essential oil of comparable yield to WT, with increased menthol and decreased menthofuran and pulegone concentrations over a 6 months period that consisted of four independent distillations and analysis.

As the levels of pulegone did not increase when *MFS* expression was reduced, further experiments were done to elucidate the link between menthofuran and pulegone. Mahmoud and Croteau (2003) overexpressed *MFS* in peppermint and found that menthofuran and pulegone concentration increased in concert suggesting that menthofuran could influence the reduction of pulegone. Although menthofuran did not inhibit PR activity, by stem feeding the transgenic peppermint menthofuran the activity of PR was decreased, accounting for the increased pulegone content in immature leaves (Mahmoud and Croteau, 2003).

These unexpected pulegone decreases and essential oil yield increase in the *MFS* antisense lines have been cause for further investigations. Using mathematical modelling and combining it with experimental hypotheses these mechanisms have been revealed further and their investigation showed that menthofuran acts as a weak inhibitor of PR (Rios-Estepa et al., 2008). By looking at essential oil synthesising secretory cells of glandular trichomes to determine if menthofuran concentrations were high enough to cause PR inhibition they showed that menthofuran was selectively retained in these cells and that exposure to abiotic stress increased the menthofuran levels further and resulted in a significant inhibition of PR (Rios-Estepa et al., 2008). Using mathematical modelling the increased essential oil yield of the *MFS* antisense plants was established to be due to the oil secretion beginning earlier. This shift in the developmental programme regulating glandular trichome development resulted in more mature glands on leaves of the same age and leaves generally having more glandular trichomes when compared to their WT counterparts (Mahmoud and Croteau, 2001; Rios-Estepa et al., 2010). Rios-Estepa et al. (2008) proposed a push-pull mechanism where the expression levels of a biosynthetic gene are elevated which, in turn, induces initiation of glandular trichomes as the reason for the increased essential oil yields in some transgenic plants. However, this hypothesis requires further testing.

Building upon the favourable essential oil profile developed by Mahmoud and Croteau (2001) and Lange et al. (2011) subjected MFS7A to multi-year field trials to confirm the favourable essential composition and increased yields, up to 69% higher than WT counterparts. Their next step was to combine this line with an additional *DXR* overexpressing construct (discussed earlier) which resulted in several transgenic lines with increases in essential oil yields of up to 78% higher than WT controls, low menthofuran and pulegone levels and high menthone and menthol levels (Lange et al., 2011).

The release of the draft sequence of *M. longifolia* and characterisation of glandular trichome specific promoters in peppermint presented some interesting opportunities for biotechnological oil modifications. Vining et al. (2017) used the *LimS* promoter to drive the expression of the *MMR* gene in peppermint plants. When compared to their WT counterparts they found a nearly fourfold increase in *MMR* transcript levels in the transgenic peppermint, a lower relative proportion of menthone (38.3 ± 2.4% vs. 43.1 ± 2.7%), and an increase in menthol (26.4 ± 0.5% vs. 22.9 ± 1.5%). The oil yields were comparable between the transgenic and WT plants (Vining et al., 2017). This exemplifies the importance of tissue specific promoters and highlights the possibilities of improved genomic resources.

A recent discovery takes us from the menthol/carvone biosynthesis pathway and into the realm of transcription factors (TFs). Information about TFs known to regulate secondary metabolism in the peltate glandular trichomes remains elusive. To this end Wang et al. (2016) isolated and functionally characterised a novel TF that is preferentially expressed in the peltate glandular trichomes of spearmint, *MsYABBY5*. YABBYs are TFs capable of acing as repressors or activators of secondary metabolites, this bifunctional nature depends on the target complex with which they interact and have also been reported to be bifunctional in the same plant (Bonaccorso et al., 2012). By generating transgenic plants overexpressing *MsYABBY5* and silencing it through RNAi they showed terpene levels decreased and increased, respectively. The transgenic *MsYABBY5* RNAi lines exhibited a significant increase in total monoterpene content, ranging from 20 to 77% and qRT-PCR showed there were no significant changes in the expression of the genes involved in carvone production and in the MEP precursor pathway (*GPPS*, *LimS*, *L6H* and *CDH*), while the overexpressing lines had a 23–52% reduction in total monoterpenes (Wang et al., 2016). This suggests that the disparity between transcript levels and metabolites can be attributed to one of the following: enhanced flux into the metabolic pathway, post-transcriptional modification or protein stability. Ectopic expression of *MsYABBY5* in *Ocimum basilicum* and *Nicotiana sylvestris* decreased secondary metabolite production in both species suggesting that this TF is likely a repressor of secondary metabolism with potential to enhance terpene production in plant glandular trichomes.

Another study on TFs in spearmint looked at the glandular trichome specific TF *MsMYB* (Reddy et al., 2017). Using RNAi they supressed the expression in spearmint and found a 2.3–4.5 fold increase in total monoterpenoid content when compared to the WT controls. Similarly to *MsYABBY5*, the overexpression of *MsMYB* in spearmint resulted in a 0.5–0.7 fold reduction in monoterpenoid content and ectopic expression of *MsMYB* in *O. basilicum* and *N. sylvestris* resulted in a reduction of terpene production but did not affect flavonoids (Reddy et al., 2017). Further analysis revealed that *MsMYB* suppresses the expression of *GPPS.LSU* through interaction with its promoter and the increased monoterpenoid production can be attributed to enhanced geranyl diphosphate production.

The above examples focus on the targetting of specific genes in the monoterpenoid biosynthesis pathway but there have been efforts to increase lipid flux in the trichomes. Hwang et al. (2020) transformed a lipid transfer protein from *N. tabacum* (NtLTP1) into *M.* × *piperita f. citrate* (Orange mint). These NtLTP1 overexpressing lines had increased trichome head diameters compared to the WT controls (Hwang et al., 2020). They also saw large increases in monoterpenoid production with limonene levels increasing 1.6 fold and 3-pinanone levels increasing over 22 fold. Overexpression of the LTP gene could prove a lucrative strategy for enhancing the production of terpenes in various *Mentha* spp.

## Effects of Plant Growth Regulators and Environmental Factors on Essential Oils

**FIGURE 2:**
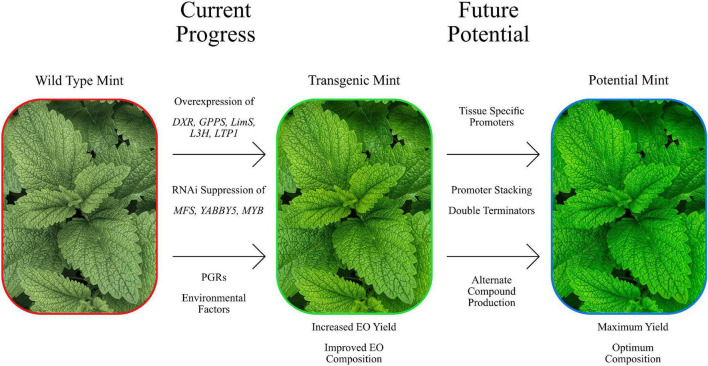
Summary image of the manipulation of *Mentha*, both present and future. Showing some of the current genetic manipulation attempts that have demonstrated efficacy at modifying EO yields compositions. Newer approaches have the potential to improve on this further.

We have discussed the numerous efforts to modify monoterpene composition and quantity above but there are also external factors whose influence can produce such effects (Summarised in Figure 2). Seasonal variation has been shown to impact the EO yield and content of *Mentha*. A study of the EO from *M. arvensis, M.* × *piperita, M. longifolia and M. spicata* grown in Pakistan showed a reduction in yield in winter harvested plants when compared to summer harvested plants (Hussain et al., 2010). A study looking at the seasonal variation of EO in *M. longifolia* grown in Tunisia (Zouari-Bouassida et al., 2018) found that winter harvested plants had a higher EO yield compared to the spring harvested, and the EO content differed between winter and spring harvested plants. Along with environmental factors, growth and developmental stage of *M.* × *piperita* have also been seen to influence the EO content. A study done on *M.* × *piperita* grown in Poland showed that the EO content and yield of field grown adult plants, *in vitro* grown plantlets and *in vitro* callus cultures differed (Bertoli et al., 2012). Interestingly, the *in vitro* grown plantlets and callus had an EO profile lacking in both pulegone and menthofuran (Croteau et al., 2005). A similar study done in *M. longifolia* showed the same pattern of *in vitro* plant biomaterial showed a EO profile lacking in pulegone and menthofuran (Bertoli et al., 2012). A study done in *M.* × *piperita* showed that the EO yield increased between early, full and late bloom, attributed to the increasing biomass (Rohloff et al., 2005). The photoperiodic treatment during the growth stage has also been shown to effect EO content. When *M. arvensis, M. citrata* and *Mentha cardiaca* were treated with varying light conditions during growth, the EO composition showed considerable variation (Farooqi et al., 1999). Short day grown plants exhibited higher concentrations of oil in their tissues compared to plants grown under normal and long day conditions. The authors proposed that this variation could be due to short day grown plants having a higher trichome density and immature leaf development. This has been noted previously in *M. arvensis*, where the neogenic peltate glands in younger leaves showed a greater accumulation of oil (Sharma et al., 2003).

Mint plants grown under drought conditions show a reduction in overall growth parameters and EO yield (Khorasaninejad et al., 2010). When *M.* × *piperita* are grown under conditions of drought stress, a significant reduction in overall growth parameters were observed. Under non-drought conditions, the EO content had a higher percentage of menthone and menthofuran. In contrast, under slight drought conditions, the EO content had a high percentage of menthol. *M. spicata* cultivated in four regions of Turkey with varying geographical and weather conditions showed variation in sesquiterpene and monoterpene compositions (Telci et al., 2010). The authors attributed these variations to regions with an elevated temperature leading to an increase in the release of monoterpenes to the atmosphere, which was found in the highest temperature and altitude region. Regions with a higher temperature showed an increase in sesquiterpene content, whilst in lower temperature regions showed an increase in monoterpene content (Telci et al., 2010). An increase in CO_2_ levels led to an increase in piperitenone oxide levels for *in vitro* grown mint cultured on basal medium containing 3% sucrose (Tisserat and Vaughn, 2001). An increase in limonene levels were also detected at 10,000 and 30,000 μmol mol^–1^ CO_2_ levels. Interestingly, soil grown plants under identical CO_2_ levels showed no appreciable change in piperitenone oxide levels, whilst no limonene was detected at all.

A study on salinity stress on *M.* × *piperita* showed that high salinity stress led to a decrease in total biomass, EO content and yield (Khorasaninejad et al., 2010). The authors speculated that these results may be due to decreased water availability or the toxicity of NaCl. Salinity stress may impose additional energy requirements on plant cells and less carbon is available for growth and flower primordial initiation, leading to less EO being synthesised. Increased salinity led to a decrease in menthofuran and an increase in menthone. A similar study showed that at elevated salinity levels, a general decrease in EO compounds were noted, including menthone and menthofuran (Li et al., 2016). The *M.* × *piperita* used in this study originated from China, so the differences in EO content after salt stress could be due to the chemotypic variation (Saeb and Gholamrezaee, 2012).

Foliar application of the cytokinins kinetin (KIN), zeatin (ZN), benzylaminopurine (BAP), and 1,3-diphenylurea (DPU) have been shown to increase the overall EO yield of several members of the *Lamiaceae* (El-keltawi and Croteau, 1987). The EO content of *M.* × *piperita* was altered by treatment with KIN and DPU, most notably an increase in the menthone content. However, treatment with BAP and ZN resulted in negligible changes to the menthone content, and a less pronounced effect on EO content overall (El-keltawi and Croteau, 1987). In a similar study, *M.* × *piperita* was treated with foliar application of various PGRs and BAP was found to be the most potent PGR in increasing menthone content (Khanam and Mohammad, 2017). Foliar application of salicylic acid (SA) caused an increase in overall oil content and yield, as well as an increase in menthol concentration and yield, and an increase in menthyl acetate yield (Khanam and Mohammad, 2017). Application of gibberellic acid (GA_3_) showed an increase in menthyl acetate content, whilst triacontanol (TRIA) caused an increase in menthone yield (Khanam and Mohammad, 2017). Foliar application of TRIA on *M. arvensis* has been shown to enhance the growth, EO yield and content of menthol, menthone, isomenthone and menthyl acetate (Naeem et al., 2011). Foliar application of TRIA in combination with gamma-irradiated carrageenan (IC) and 28-homobrassinolide (HBR) was shown to be more effective than TRIA alone, and showed an increase in menthol, menthone and menthyl acetate (Naeem et al., 2017). The authors noted that foliar application of TRIA, IC, and HBR in combination caused an increase in both chlorophyll and carotenoid content, and therefore speculated that the increase in EO yield and content was due to enhanced photosynthesis rates. The application of SA showed a significant increase in EO yield in *M.* × *piperita*, but did not significantly change the EO content (Saharkhiz and Goudarzi, 2014). SA is known to regulate a plethora of responses, including plant growth and development (Koo et al., 2020). The authors proposed that the increase in EO yield due to exogenous application of SA could be due to regulation of the plant’s energy metabolism, which might affect the stimulation of secondary metabolites in the form of EO components. The application of KIN as a foliar spray in *M. arvensis* has been shown to improve both the EO yield and content when compared to bulk KIN (Khan et al., 2022). The authors attributed these positive effects to the nanotized KIN causing an increase in the density and diameter of the peltate glandular trichomes.

Inoculating *M.* × *piperita* with plant growth promoting bacteria (PGPB) showed an increase in jasmonic acid (JA) and SA. JA and SA were shown to increase EO content up to fivefold in a dose - dependant manner. JA and SA stimulate an increased density of monoterpene accumulating glandular trichomes. Inoculation with PGPB also increases growth, however, exogenous application of JA or SA only increases EO yield, but causes a decrease in growth (Cappellari et al., 2019). Inoculation with PGPB shows an increase in overall growth, trichome density, stomatal density and secondary metabolites in *M.* × *piperita* (del Rosario Cappellari et al., 2015). Infestation of *M.* × *piperita* with the parasitic plant *Cuscuta campestris* Yunck has been shown to reduce overall growth and biomass, but increased EO yield (Sarić-Krsmanović et al., 2020). Infested *M.* × *piperita* showed an increase in the menthone content, with a decrease in menthol and pulegone content, in comparison to non-infested plants. A study on VOCs produced by various PGPBs on *M.* × *piperita* showed they can influence the composition of the EO produced (Santoro et al., 2011). The EO of *M.* × *piperita* exposed to *Pseudomonas fluorescens* showed an increase in pulegone and menthone. *M.* × *piperita* exposed to *Azospirillum brasilense* exhibited a reduction in menthol content (Santoro et al., 2011).

The use of PGRs in micropropagated apical meristem and nodal explants has been shown to influence both EO yield and content. Supplementation of *in vitro* growth media with BAP has been shown to increase the yield of EO in micropropagated *M.* × *piperita* by up to 40% (Santoro et al., 2013). Furthermore, the EO composition has been shown to be altered by the use of different combinations of PGRs (Łyczko et al., 2020). The use of cultures supplemented with 2-isopentenyladenine and indolyl-3-acetic acid showed a significant increase in menthofurolactone and decrease in limonene and eucalyptol (Łyczko et al., 2020). Menthofurolactone was originally thought to be a by-product during the oxidation of menthofuran, but was found in the EO composition of *M.* × *piperita* (Frérot et al., 2002). Clearly the effects of PGRs and environmental factors cannot be understated. While some of these are out of the control of the grower these results could provide some insight into growing practices that could alleviate some of the negative effects they encounter.

## Engineering Mint for the Future

When manipulating the biosynthetic pathways in *Mentha* there are several areas where exploitation could prove fruitful. One key area is the choice of promoter as multiple groups have seen what they suspect is co-suppression through repeated use of a single promoter (Diemer et al., 2001; Mahmoud et al., 2004). Tissier (2012) gave an excellent review of trichome specific promoters and their potential for metabolic engineering. Using strong constitutive promoters like CaMV 35S could have deleterious effects on the development and physiology of the plant through the expression of genes normally confined to the glandular trichomes. Vining et al.’s (2017) presentation of the *M. longifolia* draft genome and its use to characterise trichome specific promoters heralded a new era for tissue specific promoters in *Mentha* spp. Their use of *LimS* and *MMR* promoters to drive GUS expression and visualise high expression in the glandular trichomes of peppermint exemplifies the utility of genomic resources.

Some interesting work has been done in the area of promoters/terminators to increase expression and reduce instances of gene silencing. Transcriptional transgene silencing has been avoided by Damaj et al. (2020) who used sugarcane plants to demonstrate the potential of promoter stacking; that is, transforming a plant with multiple genetic constructs where the same coding sequence is under control of different promoters. Using this approach, the researchers managed to improve expression of a recombinant enzyme by up to 147 fold using five different promoters. Additionally, the co-expression of silencing suppressors has consistently demonstrated an improvement in gene expression by reducing post-transcriptional gene silencing. One group of researchers compared the activity of several different silencing suppressors in *N. benthamiana* and found that P19 was the most effective (Peyret et al., 2019). Furthermore, transgene expression can be improved by designing more complicated genetic constructs. Intronic regions have been shown to improve the accumulation of mRNA transcripts and are thought to improve transgene stability (Ghag et al., 2016). Some research has suggested that introns contain enhancer elements which can result in the accumulation of mRNAs by up to ten times (Egelkrout et al., 2012). Diamos and Mason (2018) demonstrated the influence of different terminator constructs on transgene expression. Using GFP as a reporter, the researchers characterised several terminators and showed that they do not act equally and can be combined in tandem to improve transgene expression. Further, the researchers also demonstrated the influence of matrix attachment regions within the constructs. Together, the use of a double terminator and matrix attachment region improved GFP production by over 60 fold in *N. benthamiana*, when compared to genetic constructs utilising only the commonly used nopaline synthase terminator. Importantly, the researchers tested these findings in both *N. benthamiana* and *Lactuca sativa* and found the terminators had differing effects between the species. This highlights the importance of optimising genetic constructs for *Mentha* species. Finally, transgene production can also be improved by affecting translation efficiency, influenced by 5′ and 3′ UTRs. Substantial engineering efforts have been made to improve UTR function in *N. benthamiana* plants, including the design of a synthetic 5′ UTR which, when used in conjunction with the Cowpea mosaic virus 3′ UTR, outperforms the commercially used Hypertrans system by approximately two-fold (Peyret et al., 2019). A combinatorial approach, utilising promoter stacking, intronic sequences, UTRs, double terminators, matrix attachment regions, and the co-expression of silencing suppressors may prove fruitful in improving secondary metabolite production in *Mentha* species. Combining this with the common syntax for type IIS assembly of plant DNA parts would simplify design, assembly and sharing of these DNA parts (Patron et al., 2015). These approaches should be investigated to alleviate the limitations at the gene expression level.

Coupling the draft *M. longifolia* sequence with the mathematical modelling of the menthol biosynthesis in peppermint could help drive target acquisition for metabolomic modifications (Rios-Estepa et al., 2010; Vining et al., 2017). Using transient expression systems like *Agrobacterium* infiltration of *N. benthamiana*, cell penetrating peptides or PEG transformation of protoplasts could prove beneficial for the testing of constructs before transforming peppermint (Niu et al., 1998, 2000; Bach et al., 2014; Norkunas et al., 2018; Fenton et al., 2020). Alternative biosynthetic routes to produce monoterpenes would include using microorganisms as biofactories. Toogood et al. (2015) used an engineered *E. coli* strain to produce menthol and neomenthol from pulegone by using two menthone dehydrogenases from peppermint, MMR and MNMR and one reductase from *N. tabacum*, NtDBR, that yielded near equivalent amounts of menthone and isomenthol. When using a single dehydrogenase, MMR or MNMR, they were able to produce highly pure menthol (79.1%) and neomenthol (89.9%), respectively, opening new avenues for producing pure compounds not produced in abundance or for (semi)-toxic chemical compounds. The same group also developed a chemoenzymatic approach to produce the four intermediates between limonene and menthone, *trans*-isopiperitenol, isopiperitenone, *cis*-isopulegone and pulegone (Cheallaigh et al., 2018). Again, this expands the capacity to generate large quantities of pure products that are usually not found in high abundance. Additional work pushed these boundaries further by using a ketosteroid isomerase from *Pseudomonas putida* (PpKSI) as a substitute for IPGI, the last enzyme in the menthol biosynthesis pathway not yet characterised in mint (Currin et al., 2018). Using engineered *E. coli* they were able to run a one pot reaction using *cis*-isopulegone as a substrate and produce menthol. Interestingly they used a robotics-driven semirational design strategy to identify a PpKSI variant with four active site mutations that conferred a 4.3 fold increase in activity over the WT. The final piece of the microbial menthol production puzzle was recently solved when Shou et al. (2021) filled in the gap between limonene and *cis*-isopulegone by replacing the microbially inefficient L3H and IPDH with a bacterial P450 monooxygenase from *P. putida* and a bacterial IPDH from *Pseudomonas aeruginosa*, respectively (Shou et al., 2021). Their system was able to produce 163.3 mg of *cis*-isopulegone from 0.54 g of limonene at a purity of 90% when analysed through GC-MS. Other work prior to limonene production has been done by Rolf et al. (2020) who transformed *E. coli* with a plasmid containing the genes encoding the enzymes in the MVA pathway and MsLimS. Using glycerol as the sole carbon source they were able to produce the highest monoterpene concentrations by a microorganism to date (Rolf et al., 2020). These results are promising and while optimisation is still needed, there are now no barriers to the microbial production of menthol, and the capacity to produce it from a single carbon source could prove a highly cost-effective biosynthesis route.

The use of microorganisms as biofactories and chemoenzymatic offers a novel way to access the intermediate monoterpenes in the menthol biosynthesis pathway. The required equipment can put this outside of the range of small holders growing mint for its essential oils for personal use or sale. In this scenario higher yielding plants would prove more fruitful and this does not necessarily need to be for menthol alone. The work done by Li et al. (2020) exemplified this when they reduced limonene expression allowing other terpenoid levels to be increased, and even producing heterologous monoterpenes (Li et al., 2020). This expands the potential to use *Mentha* as a chassis for the generation of commercially profitable compounds, like cannabidiol, that are produced by other, sometimes regulated, plants. While most of the efforts to modulate *Mentha* essential oil composition and yield have focused on the genes in or related to secondary metabolism more work could be done to target the genes involved in plant physiology, like trichome development. There have been a number of studies identifying genes associated with trichome development in *Arabidopsis*, and utilisation of these genes could prove extremely useful in *Mentha* spp. that already have a favourable oil composition (Ishida et al., 2008; Sun et al., 2015).

This review shows that substantial progress has been made in the manipulation of the biosynthetic pathways producing menthol and carvone and there is an incredible amount of potential in this space to further improve. The possibility of producing elite mint varieties with high menthol, and lower pulegone and menthofuran concentrations would not only benefit commercial growers but also smallholders in developing nations.

## Author Contributions

LF generated the figures and led the writing. All authors contributed to the article and approved the submitted version.

## Conflict of Interest

The authors declare that the research was conducted in the absence of any commercial or financial relationships that could be construed as a potential conflict of interest.

## Publisher’s Note

All claims expressed in this article are solely those of the authors and do not necessarily represent those of their affiliated organizations, or those of the publisher, the editors and the reviewers. Any product that may be evaluated in this article, or claim that may be made by its manufacturer, is not guaranteed or endorsed by the publisher.
